# Paediatric surgical trials, their fragility index, and why to avoid using it to evaluate results

**DOI:** 10.1007/s00383-022-05133-y

**Published:** 2022-05-07

**Authors:** Arne Schröder, Oliver J. Muensterer, Christina Oetzmann von Sochaczewski

**Affiliations:** 1 Klinik für Kinder- und Jugendmedizin, Klinikum Dortmund, Dortmund, Germany; 2 Kinderchirurgische Klinik und Poliklinik im Dr. von Haunerschen Kinderspital, Ludwig-Maximilians-Universität München, München, Germany; 3 Klinik und Poliklinik für Kinderchirurgie, Universitätsmedizin der Johannes-Gutenberg-Universität Mainz, Mainz, Germany; 4 Sektion Kinderchirurgie der Klinik und Poliklinik für Allgemein, Viszeral, Thorax- und Gefäßchirurgie, Universitätsklinikum Bonn, Venusberg-Campus 1, 53127 Bonn, Germany

**Keywords:** Reverse fragility index, Fragility quotient, Paediatric surgery, Uninformative metric, *S* value

## Abstract

**Background:**

The fragility index has been gaining ground in the evaluation of comparative clinical studies. Many scientists evaluated trials in their fields and deemed them to be fragile, although there is no consensus on the definition of fragility. We aimed to calculate the fragility index and its permutations for paediatric surgical trials.

**Methods:**

We searched pubmed for prospectively conducted paediatric surgical trials with intervention and control group without limitations and calculated their (reverse) fragility indices and respective quotients along with posthoc-power. Relationships between variables were evaluated using Spearman’s *ρ*. We also calculated *S* values by negative log transformation base-2 of *P* values.

**Results:**

Of 516 retrieved records, we included 87. The median fragility index was 1.5 (interquartile range: 0–4) and the median reverse fragility index was 3 (interquartile range: 2–4), although they were statistically not different (Mood’s test: *χ*^2^ = 0.557, df = 1, *P* = 0.4556). *P* values and fragility indices were strongly inversely correlated (*ρ* = − 0.71, 95% confidence interval: − 0.53 to − 0.85, *P* < 0.0001), while reverse fragility indices were moderately correlated to *P* values (*ρ* = 0.5, 95% confidence interval: 0.37–0.62, *P* < 0.0001). A fragility index of 1 resulted from *P* values between 0.039 and 0.003, which resulted in *S* values between 4 and 8.

**Conclusions:**

Fragility indices, reverse fragility indices, and their respective fragility quotients of paediatric surgical trials are low. The fragility index can be viewed as no more than a transformed *P* value with even more substantial limitations. Its inherent penalisation of small studies irrespective of their clinical relevance is particularly harmful for paediatric surgery. Consequently, the fragility index should be avoided.

## Introduction

The fragility index dates back to 2014 [1] and has been widely popularised in many specialties, [2–5] including paediatric surgery [6]. The fragility index relies on the concept of statistical significance, calculated by Fisher’s exact test, and its overturn by adding events to the group with the smallest number until statistical significance collapses. The required number of additional events is then defined as the fragility index.

Several extensions to the fragility index have been described: (1) The fragility quotient, division of the fragility index by sample size, to provide a relative measure reflecting trial sample size [7]. (2) The reverse fragility index, which is calculated by the same iterative process as in the fragility index, but aims to add events until the statistical significance collapses. Thereby, it reports the number of events that would have transformed statistically insignificant results into statistically significant ones [8]. (3) The fragility index for meta-analyses[9] and network meta-analyses [10] that allow a similar assessment, not only of trials, but their synthesis in meta-analyses.

Statistical details matter, as the controversy [11] around the readdressing [12] of the concept of posthoc-power has shown. Post-hoc power is just a transformed *P* value, [13] but this flawed [14, 15] concept has been put forward as a remedy for underpowered studies in surgery [16]. Therefore, the concept of fragility has been widely embraced due to its appealing simplicity [17]. It is marketed to simplify a complex issue, analysing trial relevance and robustness, by transformation into a single metric [18].

However, several drawbacks come with the fragility index: it can only be used to evaluate dichotomous outcomes and thereby relies on Fisher’s exact test irrespective of the test used to evaluate the results in the included trials [19]. Another issue is interpretation of the fragility index: trials can either be evaluated as fragile or as robust, but there is no clear definition of both terms [18, 20]. Consequently, terming a result fragile or robust includes a “catchy connotation” [18] that might be used to include “spin”—altered presentation of the facts—in the presentation of results of a trial, which might then disconnect its reporting from the actual results [19].

We, therefore, explored the fragility indices of paediatric surgical trials to assess the implications of the results for our specialty.

## Methods

We searched PubMed for paediatric surgical trials published through the 31st December 2019 without limitations for time, language or document type. The search was conducted on the 22nd of January 2020 and produced 516 eligible records. Following title and abstract screening, 139 records remained that were subjected to full-text evaluation. From these, 87 publications including 243 eligible comparisons were included in our analysis. Screening was conducted by two researchers in parallel and disagreements were solved by discussion and consensus. Eligible for inclusion were only prospectively conducted trials with an intervention and a separate control group. Included publications had at least one comparison with a dichotomous outcome. Included comparisons were separated by primary outcome, secondary outcome, and the remaining comparisons including those in which primary and secondary outcomes were not stated. Based on the extensions of the fragility index beyond a 1:1 allocation ratio, [5, 10, 21] we did not limit our analysis to trials with such an allocation ratio. Only two trials that did not fulfil this requirement were included. For transparency, the list of the included studies with outcomes and extracted numbers can be freely accessed from a repository [22].

Statistical analysis was conducted using R [23] (version 3.5.3) with its generic stats4-package if not stated otherwise. Fragility and reverse fragility indices were calculated as described in the introduction using the fragility index-package (version 0.1.0) [24]. The stepwise calculation of the (reverse) fragility index has been described and visualised in detail elsewhere [25]. Fragility and reverse fragility quotients were calculated by dividing them by the trial’s sample size, [7] but multiplied with 100 to avoid excessively small numbers [26]. Data are presented as medians with interquartile ranges. Correlation analyses were conducted using Spearman’s *ρ*, whose 95% confidence intervals were calculated using bootstrapping with 10,000 repetitions [27, 28] via the spearman.ci-function from the RVAideMemoire-package (version 0.9-75) [29]. Posthoc-power was calculated using the power2 × 2-function from the exact2 × 2-package (version 1.6.5) [30]. Medians were compared using Mood’s test from the RVAideMemoire-package (version 0.9–75) [31]. *P* values were Shannon-transformed by calculating their negative base-2 logarithm [32].

## Results

**Fig. 1:**
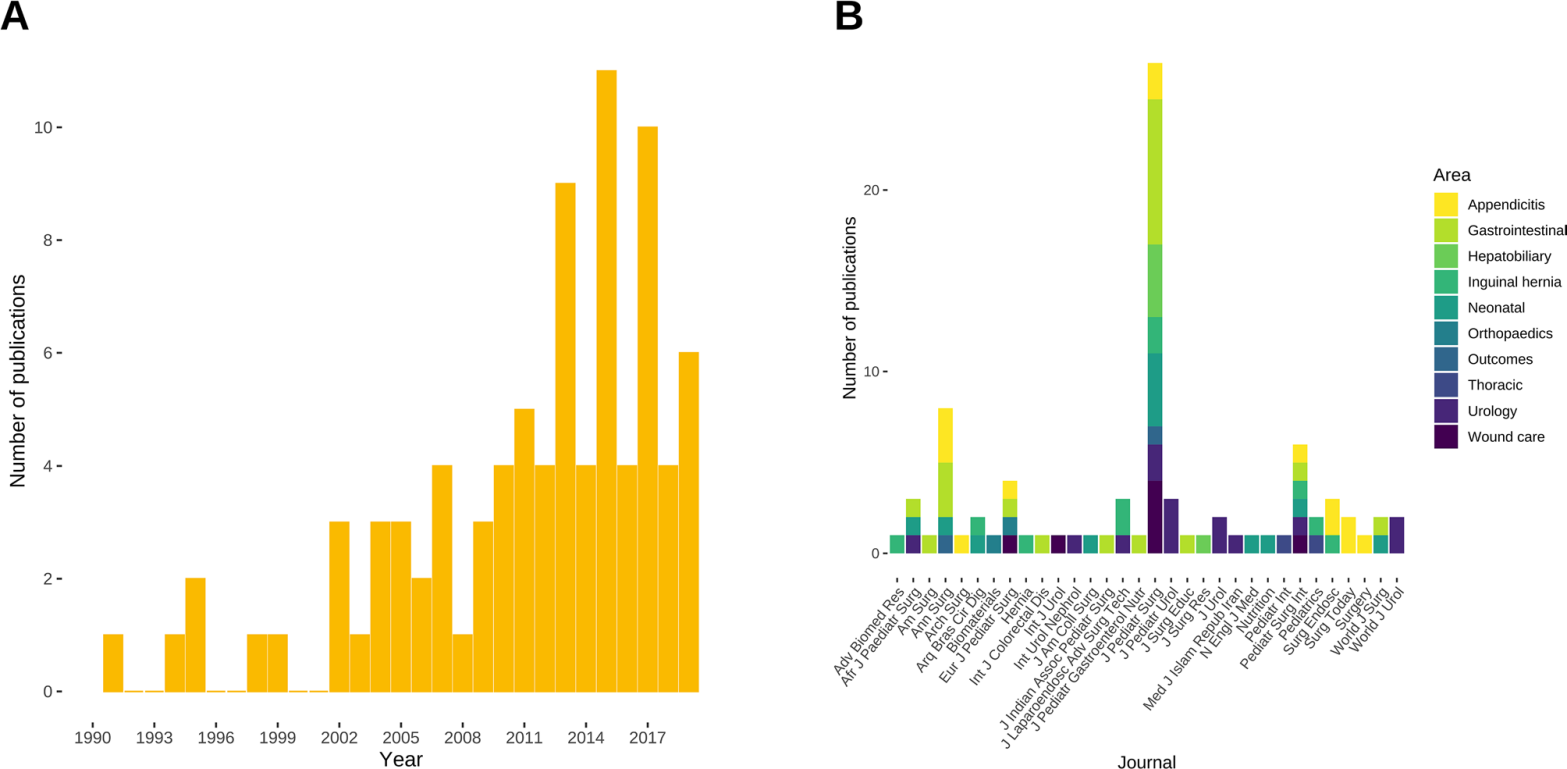
Details of the included studies. Publication year of the included studies (**A**) and publication venue by subject area (**B**)

We included 87 different publications in our analysis, the majority of which (48/87) were published between 2013 and 2019 (Fig. 1A). The Journal of Pediatric Surgery was the most frequent publication venue with 31% (27/87), followed by Annals of Surgery with 9% (8/87), and Pediatric Surgery International with 7% (6/87) of all 32 included journals (Fig. 1B). The most frequent subject areas were gastrointestinal surgery in 23% (20/87), followed by paediatric urology in 16% (14/87), and paediatric appendicitis in 15% (13/87) (Fig. 1B).

**Fig. 2:**
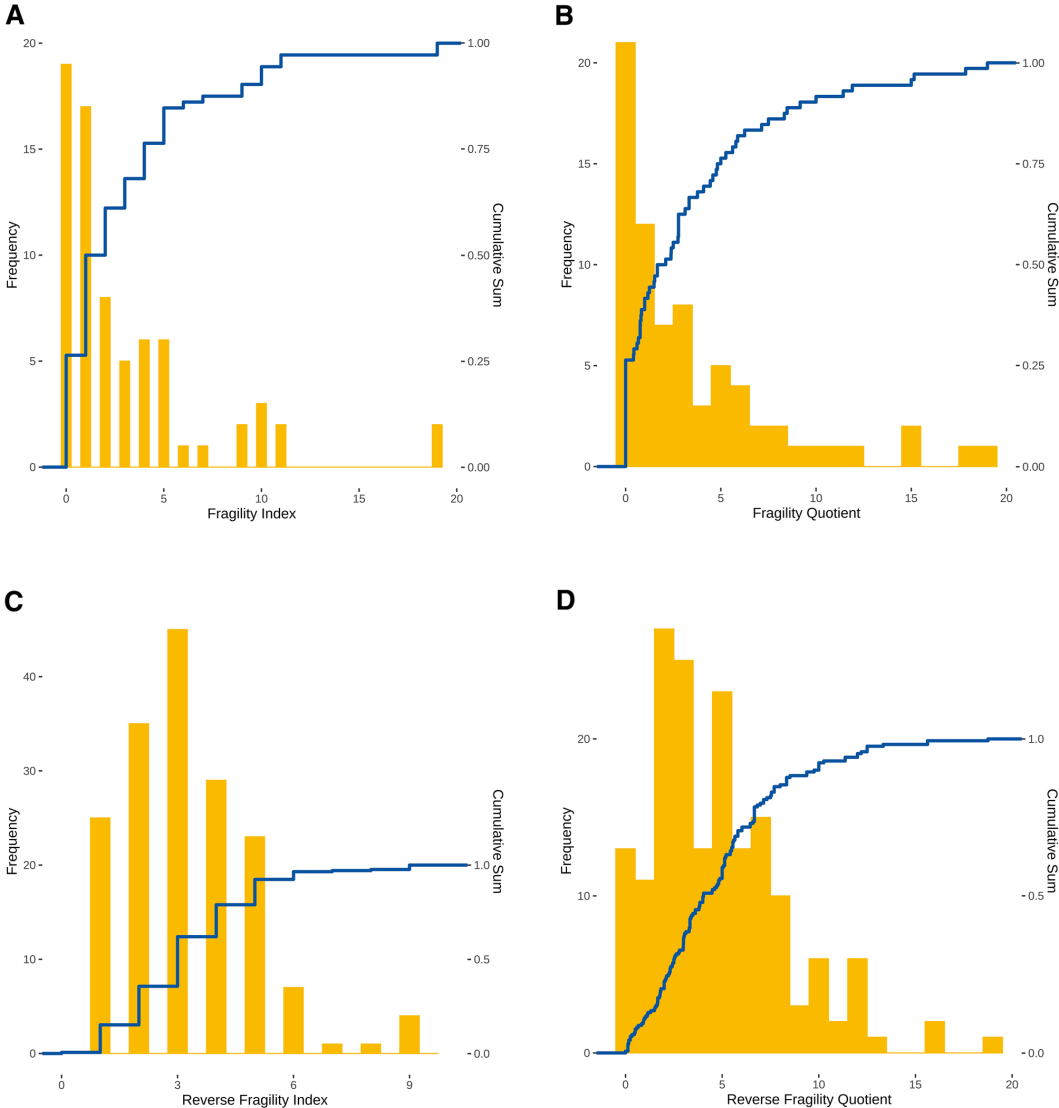
Distribution of fragility indices and fragility quotients. Histograms and cumulative sums of fragility indices (**A**), fragility quotients (**B**), reverse fragility indices (**C**), and reverse fragility quotients (**D**). Fragility quotients are displayed as percent to avoid excessively small numbers

The median fragility index of included comparisons was 1.5 (interquartile range: 0–4) (Fig. 2A) and had a median fragility quotient of 1.89% (interquartile range: 0–4.87%) (Fig. 2B). For the reverse fragility index, the median was 3 (interquartile range: 2–4) (Fig. 2C) with a corresponding median fragility quotient of 4.03% (interquartile range: 2.25–6.67%) (Fig. 2D). Dropping the two non-1:1-allocation ratio studies did neither change the median reverse fragility quotient nor its interquartile range. However, it resulted in a subtly altered interquartile range for the median fragility quotient of the reverse fragility index with an interquartile range from 2.31 to 6.67%.

**Fig. 3:**
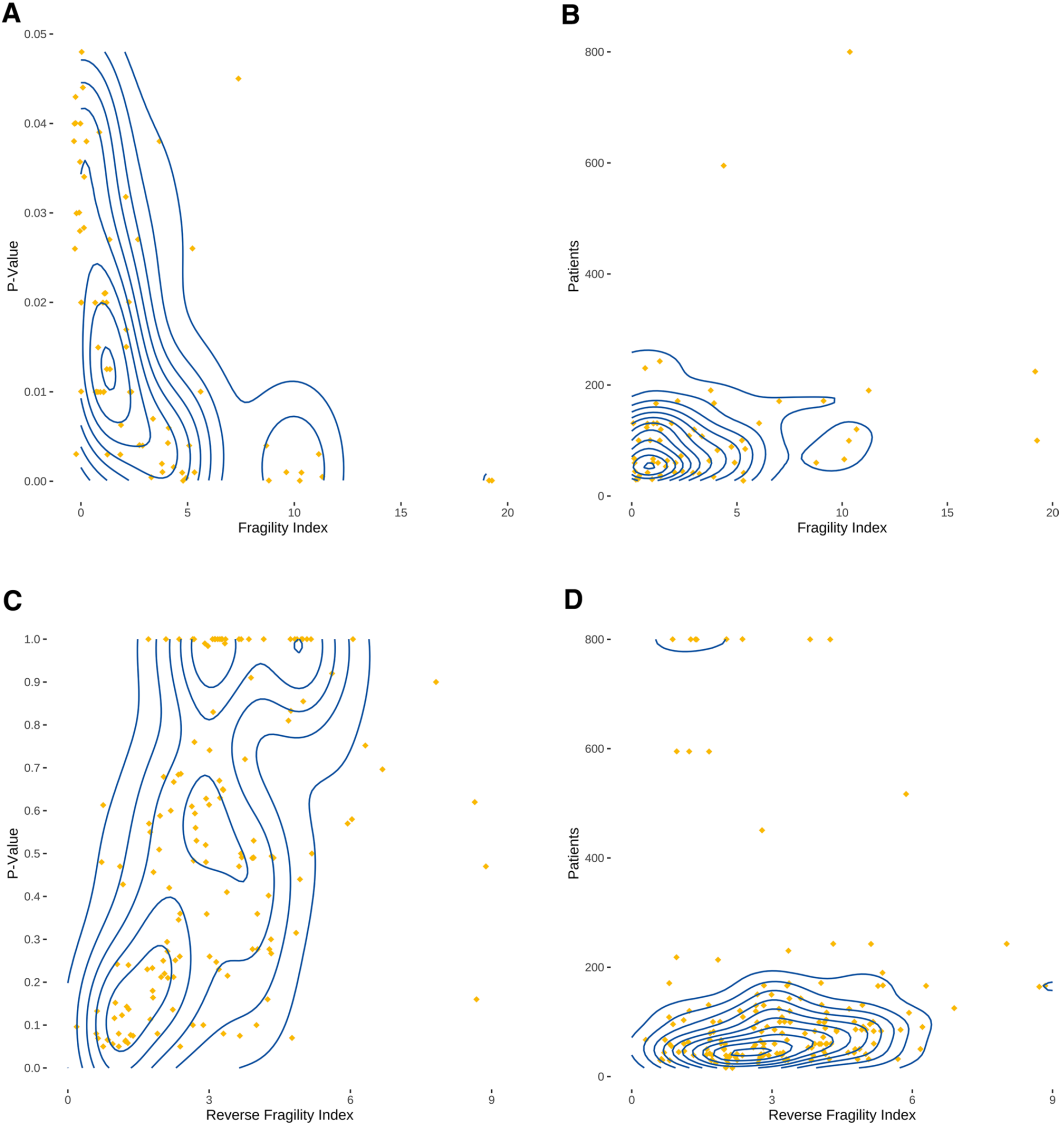
Correlation between fragility indices, *P* values, and sample size. Diamonds represent individual comparisons. Correlation analyses were conducted using Spearman’s *ρ*. **A** Fragility indices and *P* values were inversely highly correlated (*ρ* =  − 0.71, 95% confidence interval: − 0.53 to − 0.85, *P* < 0.0001). **B** Fragility indices and patient numbers were fairly correlated (*ρ* = 0.25, 95% confidence interval: 0.03–0.47, *P* = 0.0323) **C** Reverse fragility indices and *P* values were moderately correlated (*ρ* = 0.5, 95% confidence interval: 0.37–0.62, *P* < 0.0001). **D** Reverse fragility indices and patient numbers were weakly correlated (*ρ* = 0.17, 95% confidence interval: 0.007–0.32, *P* = 0.0303)

Fragility indices and *P* values were highly inversely correlated (*ρ* =  − 0.71, 95% confidence interval: − 0.53 to – 0.85, *P* < 0.0001) (Fig. 3A). In addition, the fragility indices were fairly correlated to patient numbers in the trials (*ρ* = 0.25, 95% confidence interval: 0.03–0.47, *P* = 0.0323) (Fig. 3B). Likewise, reverse fragility indices were moderately correlated to *P* values (*ρ* = 0.5, 95% confidence interval: 0.37–0.62, *P* < 0.0001) (Fig. 3C) and weakly correlated to patient numbers in the trials (*ρ* = 0.17, 95% confidence interval: 0.007–0.32, *P* = 0.0303) (Fig. 3D).

There was no difference in medians between the fragility and the reverse fragility index (*χ*^2^ = 0.557, df = 1, *P* = 0.4556), whereas the median reverse fragility quotient was larger than the median fragility quotient (*χ*^2^ = 11.035, df = 1, *P* = 0.0009).

**Fig. 4:**
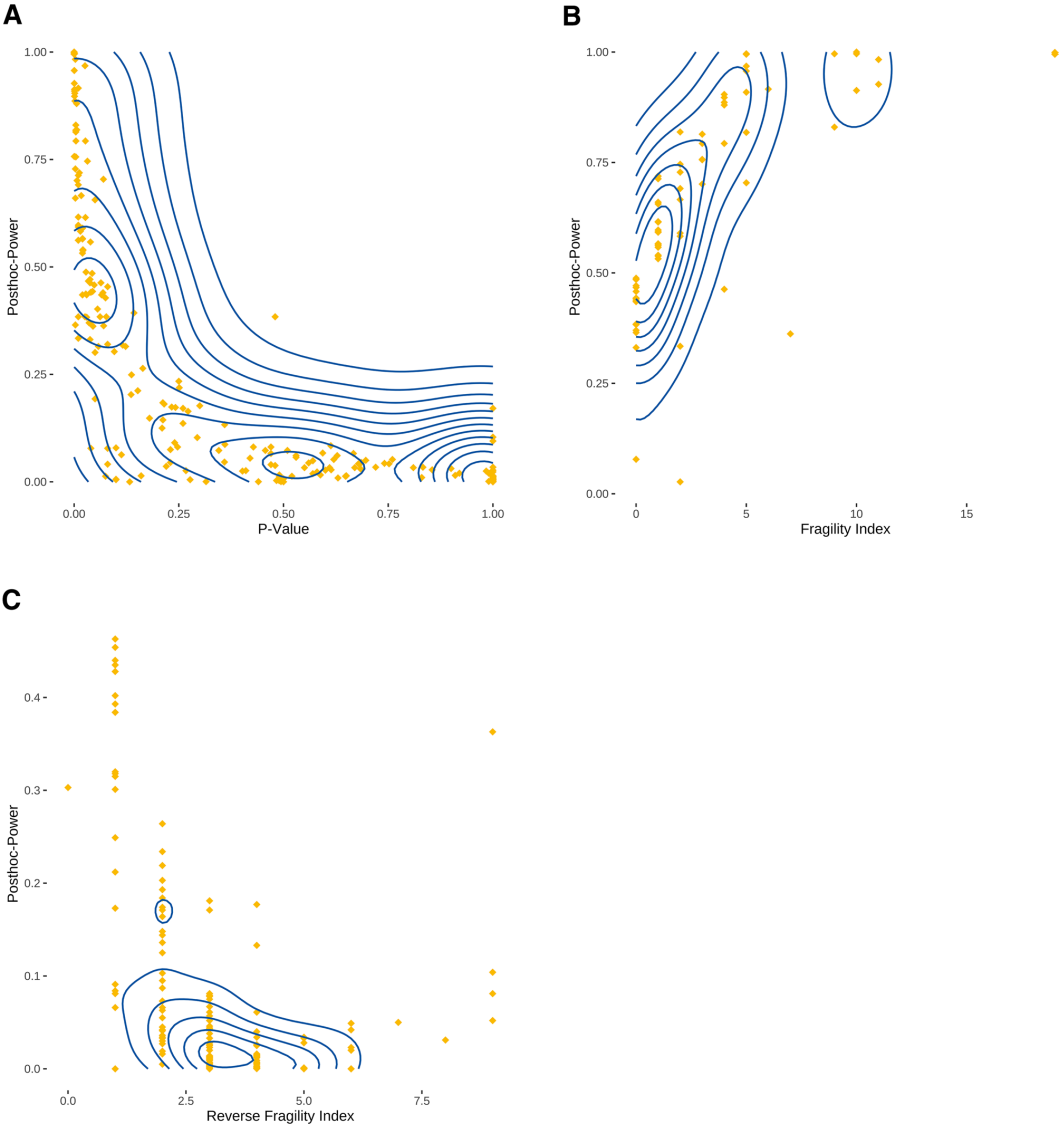
Correlation between posthoc-power, *P* values, and fragility indices. Diamonds represent individual comparisons. Correlation analyses were conducted using Spearman’s *ρ*. **A** *P* values and posthoc-power were inversely strongly correlated (*ρ* =  − 0.88, 95% confidence interval: − 0.84 to − 0.92, *P* < 0.0001). **B** Posthoc-power and fragility indices were highly correlated (*ρ* = 0.83, 95% confidence interval: 0.68–0.93, *P* < 0.0001). **C** Posthoc-power and reverse fragility indices were moderately inversely correlated (*ρ* =  − 0.66, 95% confidence interval: − 0.53 to − 0.77, *P* < 0.0001)

*P* values and posthoc-power were highly inversely correlated (*ρ* =  − 0.88, 95% confidence interval: − 0.84 to − 0.92, *P* < 0.0001) (Fig. 4A). Consequently, fragility index and posthoc-power were highly correlated (*ρ* = 0.83, 95% confidence interval: 0.68–0.93, *P* < 0.0001), too (Fig. 4B). Likewise, posthoc-power and the reverse fragility index were moderately inversely correlated (*ρ* =  − 0.66, 95% confidence interval: − 0.53 to − 0.77, *P* < 0.0001) (Fig. 4C).

The same fragility index of 1 was calculated from trials with a sample size between 30 and 243 patients and from *P* values ranging from 0.003 to 0.039. Corresponding *S* values of these *P* values are between 4.68 and 8.38 (Table 1), representing a large difference in probabilities that is not covered by the fragility index.

**Table 1:** Same fragility index can be derived from many *P* values

Study population	Fragility index	*P* value	*S* value
120	1	0.039	4.68
130	1	0.027	5.21
60	1	0.021	5.57
60	1	0.021	5.57
230	1	0.02	5.64
167	1	0.02	5.64
36	1	0.02	5.64
124	1	0.015	6.06
100	1	0.0125	6.32
100	1	0.0125	6.32
243	1	0.01	6.64
131	1	0.01	6.64
131	1	0.01	6.64
67	1	0.01	6.64
36	1	0.01	6.64
30	1	0.01	6.64
42	1	0.003	8.38

Shannon-transformation (negative log-transformation with base 2) of *P* values for all fragility indices of 1 from the included trials. The bits of information, *s*, may be interpreted as the probability of getting only heads in s unbiased coin tosses

## Discussion

Following its “invention”, the fragility index has been widely embraced by several disciplines that examined the robustness of trials in their specialty and unanimously concluded that the vast majority of trials in their field were fragile [17]. Earlier propositions to evaluate trial fragility were accompanied by a theoretical framework [33, 34]. On the contrary, the first description of the fragility index [1] was more a description than an thorough evaluation without adequate statistical simulation supporting its arguments, despite a necessity to do so [35].

Our results of a median fragility index of 1.5 with an interquartile range from 0 to 4 are similar to preceding analyses in children: in paediatric critical care, the median fragility index was 2 with an interquartile range of 1–6, [36] it was 0 with an interquartile range from 0 to 2 for preoperative androgen stimulation for hypospadias surgery, [37] and 3 with an interquartile range from 0.75 to 4.25 in paediatric appendicitis [6]. Recent results in adults are not different either: For irritable bowel syndrome, the median fragility index was 6 with an interquartile range from 0 to 25.25, [38] for proximal humerus fractures, the median fragility index was 1 with an interquartile range from 0 to 3 [39]. These results are overall similar to what has been reported before in spine surgery, otolaryngology, ophthalmology, sports surgery, and orthopaedic surgery, which all have a similar median fragility index of 2 or 3 [20].

These similar results could be expected due to the similar process in study design. Based on clinical judgement or preceding exploratory research, an effect size is defined based on the clinically relevant minimal difference [40, 41]. This is the starting point for a sample size calculation that should be able to demonstrate this difference: usually with a statistical power (1-β) of 80% and the typical α-level of 5% [41]. If the sample size is determined this way, the resulting *P* value will be slightly below the conventional cutoff of 0.05. In this context, it is important to remember the definition of the *P* value: “The probability that the chosen test statistic would have been at least as large as its observed value if every model assumptions were correct, including the test hypothesis” [42].

If the sample size of the study in question was designed to demonstrate a minimally clinically important difference with as few patients as possible, the resulting *P* value will usually not be very small: the input data are not planned to be extremely different from the null-hypothesis, but only different enough to demonstrate the effect based on conventional significance definitions. Thus, all trials will be [43] and are fragile, [20] unless the trialist has unlimited funds to expand the sample size towards infinity [44]. The fragility index ignores these principles of trial design [18, 45].

The *P* value is inherently linked to sample size: if the effect size is not exactly zero, there will always be a statistically significant *P* value, given a sufficiently large sample [40, 46]. Fisher’s exact test calculates its *P* value by the sum of two by two tables that are equal or more extreme than the observed ones [40, 47]. Consequently, an increasing sample size in a study will also increase the number of tables that are more extreme than observed, thereby reducing the *P* value. This relationship explains the correlation between fragility, reverse fragility indices, and patient numbers in the trials. This relationship could be expected based on statistical simulation [40, 48, 49]. This aspect is of particular relevance for paediatric surgery: in addition to the challenges of all surgical specialties when conducting trials, [50] paediatric surgery is further limited by the low incidence of congenital anomalies [51]. Small studies are penalised by the fragility index as it inevitably takes less events with a changed outcome to overturn statistical significance [45, 49].

This issue has been explained in detail based on an example of two hypothetical trials, [52] with the same *P* value of 0.02, but with 1 of 100 and 200 and of 4000 patients in the treatment group compared to 9 of 100 and 250 of 4000 patients in the control group who experienced a negative event. The relative risk of 0.11 in the treatment group is much more relevant than the relative risk of 0.8 in the larger trial, but the fragility index favours the larger trial [52]. The graphical depiction using consonance curves [32] emphasises this: the relative risk of the smaller trial is farther from the null and thus represents a stronger effect compared to a larger trial with the same *P* value [53].

It has been specifically discussed for paediatric surgery using the example of the highly effective foetal endoscopic tracheal occlusion for isolated congenital diaphragmatic hernia, which reports a massive clinical difference of a ten-fold risk of death in the control group with conventional treatment [53]. Nevertheless, this study could be determined fragile based on its fragility index of 3, which is caused by the small sample size that penalises the large clinical effect.

This effect is rooted in the derivation of the fragility index from the *P* value. Therefore, it has been named simply a “repackaged” *P* value [40] or a “surrogate parameter” for the *P* value [48]. The close relationship between the *P* value and the fragility index has already been described in early reports, [1–3] but it took some years until their close connection due to the derivation of the fragility index from the *P* value was demonstrated using statistical simulation [40, 48, 49]. Consequently, we observed exactly the pattern that would be expected from simulation studies: a strong inverse correlation between *P* values and fragility indices. This is also in line from what has been reported in orthopaedic trauma surgery, [54] irritable bowel syndrome, [38] paediatric appendicitis, [6] paediatrics, [55] and many more [17].

Reverse fragility indices did not behave differently: only the direction of the effect changed, because reverse fragility does not aim to remove statistical significance, but to reach it [8, 24]. Thus, we observed a strong positive correlation between *P* values and reverse fragility indices in our data set, similar to preceding assessments in other clinical specialties [8, 54]. The close relationship to the *P* value may further be depicted using posthoc-power, which simply is a function of the *P* value [13, 56]. The assessment of *P* values and posthoc-power demonstrated exactly the relationship between them predicted by statistical simulation before [11, 15]. Consequently, posthoc-power, a function of the *P* value, and both fragility indices and reverse fragility indices were correlated, similar to the results in orthopaedic surgery [48].

Due to the derivation of the fragility index from the *P* value, Porco & Lietman concluded that “Fragility retains all the problems of the *P* value, with none of the usefulness—and frankly, none of the charm” [44]. Much has been written about the problems of the *P* value, [42, 57] but the usefulness of the original version of the *P* value compared to the derived (reverse) fragility index may easily be demonstrated using Shannon-transformation. The negative base-2 logarithm of the *P* value yields the *S* value, which can ease interpretation of statistical results by nonprobabilistic measures of information [32].

The *S* value provides bits of information by which *P* values can be described by comprehensible information with known probabilities, such as a coin toss with a non-manipulated coin: [32] An *S* value of 5 would hence represent the same evidence against the null hypothesis as would five coin tosses with a non-manipulated coin that showed all heads. The same fragility index may be calculated from a widely different range of *P* values in our data and in simulation studies [48, 49]. The *S* value then exposes the weakness of this concept: the fragility index of 1 in our data corresponds to a wide range of differences in probabilities of which the highest has 91% more evidence against the null hypothesis than the smallest. The fragility index in contrast would just lump them together and label them as fragile. Consequently, the fragility index obscures important distinctions: the differences in probabilities are obvious at first sight if a sequence of four heads in four coin tosses would be equal to eight coin tosses with eight heads in a row, because they result in the same fragility index of one.

A limitation of our analysis is the inherent restriction to trials with dichotomous outcomes that is rooted within the calculation of the fragility index using Fisher’s exact test [40, 48, 49, 52]. This precludes the analysis of all numeric or time to event outcomes. Although the latter might be transformed to dichotomous data if it is simplified to the simple question if the endpoint has been reached or not, but this would be inadequate. It would strip the analysis of the important aspect of time that has passed: a patient would most likely agree that there is a relevant difference if survival is 6 months compared to 5 years. So do we and, therefore, did not conduct such analyses. Nevertheless, our search strategy seemed exhaustive enough based on similar results of the fragility index compared to other fields in both adults [1, 2, 5, 20, 38, 39] and children [6, 36, 37, 55].

Just recently, Caldwell et al. proposed an extension of the fragility index to continuous outcomes by an iterative approach: they conducted a Welch-test and changed the data point with the mean closest to the mean of the control group to the mean of the control group, which is repeated until the *P* value of the Welch-test becomes nonsignificant [58]. Apart from the fact that this method has only been tested using simulated data sets, it still retains the problem that the metric is based on the *P* value and thus inherits the problems of the conventional fragility index.

Taking into account the critique of the fragility index that it may rely on inappropriately rare outcome modifications, [59] an extension of the traditional fragility index has been proposed [60]. This extension can be generalised beyond the 2 × 2 table and also precludes unlikely modifications by taking into account the distribution of the outcome resulting in “sufficiently likely” outcome modifications [60]. However, due to its mathematical complexity, this method is beyond the scope of this manuscript. The same group has suggested that the fragility index may be used for sample size calculations in clinical trials [61]. They used two examples of coronary artery disease to illustrate their suggestions: with an estimated fragility index of 15, the sample size of one trial increased by 45% and with a fragility index of 25, the sample size of the other trial increased by 89% [61].

Apart from the financial aspect that the trialist would require much more money to conduct such a trial, it also raises an ethical issue: a trial is designed to establish its aim with as few patients as possible to minimise potential harm to those randomised to the inferior intervention [62]. It would thus be unethical to further randomise patients to an intervention that is known—due to the mandatory intermediate review—to be inferior, just to achieve certain levels of the fragility index [62]. For this specific aim, the adaptive trial design has been developed to avoid both underpowered trials and randomising patients to futile treatments [63]. Such a trial would always be considered fragile based on the fragility index alone, [62] which emphasises that it may not be used to determine trial sample size.

Besides this quite technical line of argument, the use of the fragility index in paediatric surgery is rather limited per se. It could have only been used on results of (randomised) controlled, prospectively conducted trials with a dichotomous outcome and thus not applicable to the vast majority of research data in paediatric surgery. Moreover, our study addresses only one of the many potential errors that can be made during the scientific process: [64] The wrong interpretation of the *P* value and its derivatives are potentially harmful, but the focus on them should not obscure other problems, such as poor study design and data collection or the “hunt for significance” [65]. They also would inevitably lead to poor results or as it had been pointed out in the context of meta-analyses already in 2001 with the famous proverb “garbage in, garbage out” [66]. Nonetheless, the still ongoing discussion about the fragility index might emphasise the point of critical assessment of research methods per se: “Perhaps the most valuable contribution of the fragility index is that its shortcomings and inconsistencies have inspired scientists to reflect deeply on what it truly means […],” [52] or more pointed “So it is perhaps odd that, while we allow a doctor to conduct surgery only after years of training, we give SPSS^®^ (SPSS, Chicago, IL) to almost anyone”[67].

Fragility indices, reverse fragility indices, and their respective fragility quotients of paediatric surgical trials are low. The fragility index can be viewed as no more than a transformed *P* value with even more substantial limitations. Its inherent penalisation of small studies irrespective of their clinical relevance is particularly harmful for paediatric surgery. Consequently, the fragility index should be avoided.

## Data Availability

The data sets generated and analysed during the current study are available in the Zenodo repository (https://doi.org/10.5281/zenodo.4883231) [22].
